# A survey of the attitudes, beliefs and knowledge about medical cannabis among primary care providers

**DOI:** 10.1186/s12875-019-0906-y

**Published:** 2019-01-22

**Authors:** Lindsey M. Philpot, Jon O. Ebbert, Ryan T. Hurt

**Affiliations:** 1 Department of Medicine, Mayo Clinic, 200 1st Street SW, Rochester, MN 55905 USA; 2 Robert D. and Patricia E. Kern Mayo Clinic Center for the Science of Health Care Delivery, Mayo Clinic, 200 1st Street SW, Rochester, MN 55905 USA

**Keywords:** Cannabis, Medical marijuana, Health care surveys, Primary care physicians, Primary care

## Abstract

**Background:**

Healthcare providers play a critical role in facilitating patient access to medical cannabis. However, previous surveys suggest only a minority of providers believe that medical cannabis confers benefits to patients. Significant new knowledge about the potential benefits and harms of medical cannabis has recently emerged. Understanding current attitudes and beliefs of providers may provide insight into the ongoing challenges they face as states expand access to medical cannabis.

**Methods:**

We conducted an electronic survey of primary care providers in a large Minnesota-based healthcare system between January 23 and February 5, 2018. We obtained information about provider characteristics, attitudes and beliefs about medical cannabis, provider comfort level in answering patient questions about medical cannabis, and whether providers were interested in receiving additional education.

**Results:**

Sixty-two providers completed the survey (response rate 31%; 62/199). Seventy-six percent of respondents were physicians and the average age was 46.3 years. A majority of providers believed (“strongly agree” or “somewhat agree”) that medical cannabis was a legitimate medical therapy (58.1%) and 38.7% believed that providers should be offering to patients for managing medical conditions. A majority (> 50%) of providers believed that medical cannabis was helpful for treating the qualifying medical conditions of cancer, terminal illness, and intractable pain. A majority of providers did not know if medical cannabis was effective for managing nearly one-half of the other state designated qualifying medical conditions. Few believed that medical cannabis improved quality of life domains. Over one-third of providers believed that medical cannabis interacted with medical therapies. One-half of providers were not ready to or did not want to answer patient questions about medical cannabis, and the majority of providers wanted to learn more about it.

**Conclusions:**

Healthcare providers generally believe that medical cannabis is a legitimate medical therapy. Provider knowledge gaps about the effectiveness of medical cannabis for state designated qualifying conditions need to be addressed, and accurate information about the potential for drug interactions needs to be disseminated to address provider concerns. Clinical trial data about how medical cannabis improves patient quality of life domains is desperately needed as this information can impact clinical decision-making.

**Electronic supplementary material:**

The online version of this article (10.1186/s12875-019-0906-y) contains supplementary material, which is available to authorized users.

## Background

Cannabis is a term used for pharmacologic agents derived from plants belonging to the genus *Cannabis* [1]. The US Comprehensive Drug Abuse Prevention and Control Act of 1970 lists cannabis as a Drug Enforcement Agency schedule I drug with use prohibited for any purpose [2, 3]. However, 29 US states and the District of Columbia have comprehensive programs authorizing cannabis for use in specific medical conditions [4]. With the exception of the District of Columbia, all states specify the qualifying medical conditions for which cannabis can be used [5], and most states require healthcare providers to be registered with the state in order to certify patients for qualifying medical conditions. Cannabis is supplied to patients through state designated medical cannabis dispensaries. Routes of medical cannabis self-administration vary widely by state with many providing capsules, oil, and vaporizing liquid [4]. Smoked medical cannabis is prohibited by many states as are edibles. In addition to the comprehensive programs, another 17 states have limited access cannabis laws that limit the product to a low tetrahydrocannabinol (THC) and high cannabinoid content. The majority of these limited access cannabis laws are limited to seizure indications.

The National Academies of Sciences, Engineering, and Medicine (NASEM) conducted and recently published a comprehensive review of the medical literature on the health effects of cannabis and cannabinoids [6]. NASEM concluded that there is “conclusive and substantial evidence” that medical cannabis is effective for alleviating chronic pain, chemotherapy-induced nausea and vomiting, and spasticity associated with multiple sclerosis [7]. NASEM also concluded that there is “substantial” evidence for an association between cannabis smoking and respiratory disease, and between cannabis use and motor vehicle collisions, lower birth weight offspring, and schizophrenia or other psychosis.

However, differences between cannabis products used in clinical trials and products available to patients in different states create uncertainty for healthcare providers attempting to make decisions and educate patients about medical cannabis. Data informing NASEM conclusions about the efficacy of cannabis for chronic pain, for example, included studies evaluating smoked cannabis, not available in some state dispensaries, and prescription synthetic cannabinoids, not available in any state dispensaries [8]. Different products and modes of delivery would be reasonably expected to have different benefit and risk profiles. Awareness of or concern about clinically important drug interactions between medical cannabis and other medical therapies may also heighten provider uncertainty [9].

A recent survey demonstrated that a majority of patients believe that cannabis can be used to treat pain and anxiety and reduce the amount of opioid medication used for pain management [10]. An online survey revealed that a majority of patients and the public believed sufficient safety and efficacy data exist to justify use of medical cannabis for epilepsy while only a minority of epileptologists and general neurologists believed this [11]. In a survey of Colorado primary care providers, a minority endorsed that medical cannabis conferred physical and mental benefits to patients [12]. Since the Colorado survey, several large systematic reviews [8, 13, 14] and the NASEM report [6] have been published and more states have legalized medical cannabis. We were unable to identify any published surveys that have assessed the degree to which primary care healthcare providers believe medical cannabis is beneficial for the state designated qualifying medical conditions specific to the state of their medical practice.

In May 2014, Minnesota became the 22nd US state to create a medical cannabis comprehensive program, and enrolled patients had access to extracted cannabis products in liquid or oil form beginning on July 1, 2015. As of October 2017, 7022 Minnesotans have been certified through the Minnesota Medical Cannabis Program [15]. Qualifying conditions for medical cannabis in Minnesota currently include cancer associated with severe/chronic pain, nausea or severe vomiting, or cachexia or severe wasting; glaucoma; HIV/AIDS; Tourette syndrome; amyotrophic lateral sclerosis (ALS); seizures, epilepsy; severe and persistent muscle spasms, including those characteristic of multiple sclerosis (M.S.); inflammatory bowel disease, including Crohn’s disease; terminal illness with a probable life expectancy of less than one year; intractable pain; post-traumatic stress disorder, obstructive sleep apnea, and autism.

Advancing our understanding of the attitudes, beliefs, and knowledge of practicing clinicians may identify ongoing barriers, biases and knowledge gaps relating to medical cannabis. In order to address this issue, we conducted an electronic survey of primary care providers in a health system in Midwest United States.

## Methods

### Setting and study population

Located within Southeastern Minnesota, Mayo Clinic manages the care for approximately 152,000 patients residing in and around Olmsted County, Minnesota through a longitudinal care practice comprised of Internal Medicine and Family Medicine providers. Primary care providers within Mayo are responsible for the longitudinal care of our local and community-based population. Longitudinal care comprises preventive and wellness care, management of chronic health conditions, addressing acute issues that arise, and coordination of care with our specialty practices as needed. A link to participate in an anonymous, web-based survey was sent to the institutional email addresses of a convenience sample of the 199 physicians (Medical Doctor, MD; Doctor of Osteopathy, DO; Bachelor of Medicine, Bachelor of Surgery (MBBS)), nurse practitioners (NP), and physician assistants (PA) responsible for the primary care of these patients. Respondents were allowed two weeks to complete the survey (January 23, 2018 through February 5, 2018). One initial invitation, and two reminder E-mails were sent to participants to complete the survey during the two week period.

The study was reviewed by the Mayo Institutional Review Board and determined to be exempt from the requirement for IRB approval (45 CFR 46.101b, item 2) including waiver of informed consent.

### Questionnaire

The new survey tool was developed by first defining domains of interest (knowledge, attitudes, and beliefs) (Additional file 1). Questions in these domains were informed by qualitative data from patient surveys reported through the Minnesota Medical Cannabis Program. Questions were pilot tested and refined. Survey items were placed on 3- or 5-point Likert scales to garner patient agreement or perceived degree of helpfulness or frequency, based on the individual survey item. The 16-item survey was designed to address the following areas.

#### Provider characteristics and knowledge

We assessed clinician licensure (MD/DO/MBBS, NP/PA), primary care departments (Internal Medicine or Family Medicine), age, years in practice, and gender. We also assessed whether clinicians are registered to certify patients for medical cannabis and, if so, how many patients they have certified. In addition, we asked whether clinicians felt confident about answering patient questions about medical cannabis and whether the clinicians were interested in learning more about the topic.

#### Provider attitudes and beliefs regarding medical Cannabis as a therapy in general

We first assessed clinician attitudes about the legitimacy of medical cannabis as a medical therapy in general and beliefs regarding overall effectiveness. We also assessed provider beliefs that patients were using cannabis illegally to treat symptoms outside of the state program and their beliefs on the effectiveness of state designated medical cannabis as opposed to illegal cannabis used by patients. Furthermore, we asked about the perceived level of difficulty providers felt in enrolling and maintaining patients on medical cannabis therapy through the state program.

#### Provider beliefs about specific disease and symptom control and perceived benefits to patient quality of life

We assessed provider beliefs in how helpful medical cannabis is a medical therapy for the 13 state designated medical conditions, how helpful medical cannabis is as a medical therapy to control individual symptoms, and to what extent medical cannabis positively impacts the quality of life of patients. We assessed the following symptoms: pain, seizures, nausea and vomiting, appetite, anxiety, depression, insomnia, weight loss, and tics. Quality of life (QOL) measures included: physical functioning, energy level, mood, enjoyment of life, social engagement, ability to work, and sense of hope.

### Statistical analyses

We summarized provider characteristics using summary statistics. Respondent count and proportion were calculated based on total respondents per question, and the number skipping a question was not included in the denominator. All data management and statistical analyses were performed using Statistical Analysis Software (SAS) Version 9.3 (Cary, North Carolina).

## Results

### Provider characteristics and knowledge

Sixty-two providers completed the survey (response rate 31%; 62/199). Seventy-six percent of respondents were physicians and the average age was 46 years (Table 1). A minority (27.4%) was registered to certify patients for medical cannabis, but one-half reported having patients who had been certified for medical cannabis. The mean number of patients certified by certifying providers was 4. One-half of providers were not ready or did not want to answer patient questions about medical cannabis, and over three-quarters of providers were interested in learning more about the topic.

**Table 1:** Provider Characteristics and Knowledge (*N* = 62)

Provider Licensure, n (%)	
Physician (MD, DO, MBBS)	45 (75.6)
Advanced Practice Professional (NP, PA)	17 (27.4)
Provider Specialty, n (%)	
Internal Medicine	33 (53.2)
Family Medicine	29 (46.8)
Age, years	
Mean (SD)	46.3 (11.8)
Median (IQR)	43.5 (36.0, 58.0)
Range	27.0–65.0
Gender, n (%)	
Female	26 (42.6)
Male	35 (57.4)
Registered to certify patients for medical cannabis, n (%)	
Yes	17 (27.4)
No	45 (75.6)
Number of patients certified (*n* = 13 providers report certifying)	
Mean (SD)	3.7 (3.7)
Median (IQR)	3.0 (2.0, 4.0)
Range	1.0–15.0
Report of ever certifying a patient, n (%)	
Yes	16 (25.8)
No	46 (74.2)
Report of having patients who have been certified, n (%)	
Yes	20 (50.0)
No	20 (50.0)
Interest in learning more about medical cannabis, n (%)	
Yes	48 (77.4)
No	14 (22.6)
Ready to answer questions about medical cannabis, n (%)	
Yes	31 (50%)
No	28 (45.2%)
Do not want to answer questions about cannabis	3 (4.8%)

*SD* Standard Deviation, *IQR* Interquartile Range

### Provider attitudes and beliefs regarding medical Cannabis as a therapy in general

A majority of providers believed (“strongly agree” or “somewhat agree”) that medical cannabis was a legitimate medical therapy and over one-third believed that providers should be offering it to patients for managing medical conditions (Table 2). A majority thought it could effectively treat symptoms associated with medical conditions, but 38.7% believed that medical cannabis has significant interactions with medical therapies. A minority thought that the Minnesota state patient certification process was a barrier to enrollment. Almost all providers were aware of some of their patients using cannabis illegally to manage symptoms or medical conditions. A majority thought the cannabis acquired through the state was safer than that obtained illegally although only a minority thought it was more effective.

**Table 2:** Provider Attitudes and Beliefs Regarding Medical Cannabis in General, N (%)

	Strongly agree	Somewhat agree	Neither agree nor disagree	Somewhat disagree	Strongly disagree
Medical cannabis is a legitimate medical therapy.	13 (21.0)	23 (37.1)	18 (29.0)	3 (4.8)	5 (8.1)
Medical providers should be offering medical cannabis for managing medical conditions.	10 (16.1)	14 (22.6)	20 (32.3)	12 (19.3)	6 (9.7)
Medical cannabis has significant interactions with medical therapies.	5 (8.1)	19 (30.6)	27 (43.6)	9 (14.5)	2 (3.2)
Medical cannabis can effectively treat symptoms associated with medical conditions.	13 (21.0)	24 (38.7)	20 (32.3)	3 (4.8)	2 (3.2)
The process to certify patients in the medical cannabis program prevents me from enrolling patients.	5 (8.2)	10 (16.4)	15 (24.6)	16 (26.2)	15 (24.6)
I am aware that patients use cannabis illegally to treat symptoms or medical conditions.	41 (66.1)	17 (27.5)	2 (3.2)	0 (0.0)	2 (3.2)
Medical cannabis through the state of Minnesota is safer than cannabis that patients use illegally.	21 (33.9)	17 (27.4)	19 (30.6)	3 (4.8)	2 (3.2)
Medical cannabis through the state of Minnesota is more effective than cannabis that patients use illegally.	6 (9.8)	8 (13.1)	37 (60.7)	9 (14.8)	1 (1.6)

### Provider beliefs about specific disease and symptom control and perceived benefits to patient quality of life

A majority of providers believed that medical cannabis was helpful for treating the qualifying medical conditions of cancer, terminal illness, and intractable pain (Table 3). One-half of providers believed that it was effective for muscle spasms, including those characteristic of M.S.. Forty-two percent believed it was effective for seizures, and over one-third believed that medical cannabis was effective for glaucoma. A majority of providers did not know if it was effective for managing Tourette syndrome, amyotrophic lateral sclerosis (ALS), inflammatory bowel disease, post-traumatic stress disorder, obstructive sleep apnea, or autism. A majority of providers believed that medical cannabis was effective for managing clinical symptoms of pain, nausea and/or vomiting, and anxiety. Over one-third believed it was effective for managing insomnia. A minority of providers believed that medical cannabis improved patient physical functioning, energy, mood, enjoyment of life, social engagement, ability to work, and sense of hope.

**Table 3:** Provider Beliefs about Specific Diseases and Symptom Control, and Perceived Benefits to Patient Quality of Life, N (%)

	Positive		Neutral	Negative		Don’t Know
	1	2	3	4	5	6
Qualifying Medical Conditions (H)						
Cancer associated with severe/chronic pain, nausea or severe vomiting, or cachexia or severe wasting	20 (32.3)	29 (46.8)	2 (3.2)	0 (0.0)	2 (3.2)	9 (14.5)
Glaucoma, missing = 1	3 (4.9)	19 (31.2)	7 (11.5)	0 (0.0)	2 (3.3)	30 (49.2)
HIV/AIDS	4 (6.5)	12 (19.4)	12 (19.4)	0 (0.0)	4 (6.5)	30 (48.4)
Tourette Syndrome	3 (4.8)	13 (21.0)	10 (16.1)	1 (1.6)	2 (3.2)	33 (53.2)
Amyotrophic Lateral Sclerosis (ALS)	5 (8.1)	12 (19.4)	8 (12.9)	0 (0.0)	3 (4.8)	34 (54.8)
Seizures, including those characteristic of Epilepsy	8 (12.9)	18 (29.0)	8 (12.9)	1 (1.6)	2 (3.2)	25 (40.3)
Severe and persistent muscle spasms, including those characteristic of Multiple Sclerosis	8 (12.9)	23 (37.1)	7 (11.3)	0 (0.0)	3 (4.8)	21 (33.9)
Inflammatory bowel disease, including Crohn’s disease	3 (4.8)	8 (12.9)	12 (19.4)	1 (1.6)	5 (8.1)	33 (53.2)
Terminal illness, with a probable life expectancy of less than one year	14 (22.6)	23 (37.1)	4 (6.5)	1 (1.6)	2 (3.2)	18 (29.0)
Intractable pain	14 (22.6)	28 (45.2)	3 (4.8)	2 (3.2)	4 (6.5)	11 (17.7)
Post-Traumatic Stress Disorder, missing = 2	5 (8.3)	10 (16.7)	11 (18.3)	1 (1.7)	2 (3.3)	31 (51.7)
Obstructive Sleep apnea	1 (1.6)	2 (3.2)	13 (21.0)	4 (6.5)	8 (12.9)	34 (54.8)
Autism	2 (3.2)	1 (1.6)	11 (17.7)	4 (6.5)	7 (11.3)	37 (59.7)
Symptoms (H)						
Pain	12 (19.4)	37 (59.7)	2 (3.2)	0 (0.0)	4 (6.5)	7 (11.3)
Seizures	10 (16.1)	20 (32.3)	9 (14.5)	2 (3.2)	2 (3.2)	19 (30.7)
Nausea and/or vomiting	9 (14.5)	33 (52.3)	5 (8.1)	3 (4.8)	2 (3.2)	10 (16.1)
Muscle spasms	4 (6.5)	20 (32.3)	15 (24.2)	0 (0.0)	3 (4.8)	20 (32.3)
Anxiety	8 (12.9)	28 (45.2)	6 (9.7)	6 (9.7)	4 (6.5)	10 (16.1)
Depression	3 (4.8)	8 (12.9)	7 (11.3)	20 (32.3)	11 (17.7)	13 (21.0)
Sleeplessness	4 (6.5)	17 (27.4)	12 (19.4)	3 (4.8)	6 (9.7)	20 (32.3)
Weight loss	5 (8.1)	7 (11.3)	12 (19.4)	6 (9.7)	10 (16.1)	22 (35.5)
Tics	3 (4.8)	13 (21.0)	9 (14.5)	2 (3.2)	2 (3.2)	33 (53.2)
Quality of Life (E)						
Physical functioning	4 (6.5)	10 (16.1)	18 (29.0)	10 (16.1)	4 (6.5)	16 (25.8)
Energy level	2 (3.2)	4 (6.5)	15 (24.2)	9 (14.5)	10 (16.1)	22 (35.5)
Mood	3 (4.8)	10 (16.1)	17 (27.4)	10 (16.1)	5 (8.1)	17 (27.4)
Enjoyment of life	3 (4.8)	15 (24.2)	18 (29.0)	5 (8.1)	3 (4.8)	18 (29.0)
Social engagement (visiting with friends and family), missing = 1	4 (6.6)	10 (16.4)	16 (26.2)	9 (14.8)	3 (4.9)	19 (31.2)
Ability to work	2 (3.2)	9 (14.5)	12 (19.4)	11 (17.7)	8 (12.9)	20 (32.3)
Sense of hope, missing = 1	4 (6.6)	11 (18.0)	14 (23.0)	9 (14.8)	4 (6.7)	19 (31.2)

(H) Helpfulness Scale (1, Very helpful; 2, Somewhat helpful; 3, Neither helpful nor not helpful; 4, Somewhat not helpful; 5, Not at all helpful; 6, Don’t know)

(E) Extent Scale (1, A great deal; 2, Quite a bit; 3, Somewhat; 4, Very little; 5, Not at all; 6, Don’t know)

## Discussion

We observed that although primary care healthcare providers in a state with an established program generally believe that medical cannabis is a legitimate medical therapy and safer than illegal cannabis, significant knowledge gaps existed about effectiveness related to state designated qualifying medical conditions and one-half were not prepared to answer patient questions about it. Most providers indicated they were aware that some of their patients were using cannabis illegally to treat medical conditions, but few believed that medical cannabis improved patient quality of life domains. A significant proportion of providers endorsed that medical cannabis had potential interactions with medical therapies. Most providers were interested in learning more about medical cannabis.

Only three of thirteen Minnesota state designated qualifying medical conditions had a majority of providers endorsing that medical cannabis was an effective management strategy for them. However, more than one-half of providers believed that medical cannabis was effective for the management of nausea/vomiting, muscle spasms including those characteristic of M.S., and pain, three conditions for which the best evidence exists [6]. Knowledge gaps in the literature exist regarding the effectiveness of medical cannabis for different medical conditions, and data available supporting the potential efficacy of medical cannabis for state designated qualifying medical conditions varies substantially. For example, available data suggests cannabinoid use is associated with only short-term reductions in intraocular pressure [16]; the National Academies of Sciences, Engineering, and Medicine (NASEM) concluded that limited evidence suggests that they are *ineffective* for the treatment of glaucoma [6], one of the most common qualifying medical conditions among state medical cannabis programs [17]. States interested in maintaining medical cannabis laws for medical indications could consider a more evidence-based approach when adding additional qualifying conditions. This approach may increase the perceived legitimacy of medical cannabis for qualifying conditions among certifying clinicians and increase the likelihood that providers would consider medical cannabis as part of a comprehensive treatment strategy.

Most providers did not endorse that they believed medical cannabis improved aspects of patient quality of life (QOL). Systematic reviews have demonstrated that cannabinoids improve QOL among patients taking them for chronic neuropathic pain [18]. This particular review included studies evaluating dronabinol (THC), nabilone (THC biosimilar), and nabiximols (THC and cannabidiol, CBD). Such information is informative for both patients and providers in Minnesota as products available through the state program consist exclusively of THC and/or CBD. However, exceptionally few studies on medical cannabis have collected QOL measures [6] making it difficult for providers to be informed about potential benefit in these domains. Researchers should be encouraged to include quality of life measures, such as the SF-36 [19], especially in prospective studies as this questionnaire is publicly-available, validated, and easily collected. Patient QOL considerations have significant impact on provider clinical decision-making [20]. Adding QOL measures to an assessment battery along with the outcomes of interest (e.g., pain and spasticity) should not be viewed as overly burdensome and will critically advance the science.

We observed that over one-third of providers believed that medical cannabis has significant interactions with medical therapies. Providers need to be informed of potential interactions between medications they prescribe and products obtained from state dispensaries. Even when products contain only THC and CBD as constituents, providers and allied health professionals have little understanding of the concentration of these cannabinoids in the formulations patient receive from the state dispensary and this information is not loaded into electronic medical records. As a result, software automatically flagging drug interactions cannot assist prescribers. To address this concern, clinicians may stop medical cannabis in order to avoid these interactions when starting new medications, a problem that may be exacerbated in states making available smoked cannabis which contains over 100 phytocannabinoids (i.e., natural plant products) [21]. Although the evidence for significant drug interactions with THC and CBD is rare, THC and CBD are metabolized by CYP1A2, CYP2C9, CYP2C19, and CYP3A4 and serum concentrations of these molecules may theoretically increase with medications that inhibit or induce these enzymes [22]. Greater information sharing between dispensaries, patients and providers regarding the dosing of medical cannabis and the relative concentrations of medical cannabis constituent components present opportunities to improve patient safety.

We observed that 50% providers were not ready or did not want to answer patient questions about medical cannabis. This is consistent with previous research among US residents and fellows observing that only 35.3% of respondents felt ready to answer patient questions about cannabis [23]. Reassuringly, we found that over three-quarters of providers were interested in learning more about medical cannabis. Previous surveys have reported a strong desire among providers to obtain and support additional education on medical cannabis [12, 24]. Given the expansion of medical cannabis in the US, medical education and continuing medical education needs to include curricula on cannabinoids or these profound knowledge gaps will put patients and providers at risk.

The strengths of our survey include that it was conducted among primary care providers in a large, integrated healthcare system in a state where medical cannabis laws have been present for two years, we assessed beliefs about the effectiveness of medical cannabis for state designated qualifying conditions, and we observed a high response rate. Our study is limited by sample size and generalizability as questions were referential to the Minnesota state cannabis program, but this program is very similar to other state programs.

## Conclusions

Providers generally believe that medical cannabis is a legitimate medical therapy. Significant opportunities exist to: 1) close knowledge gaps for clinicians through the collection and dissemination of information about the effectiveness of medical cannabis for state qualifying conditions; 2) alleviate concerns about drug interactions by exploring opportunities for information sharing between dispensaries and traditional medical practices; and 3) expand the knowledge base about how medical cannabis impacts patient QOL.

## Additional file

Additional file 1: Provider Cannabis Survey. Survey Tool Sent to Providers about Medical Cannabis. (DOCX 33 kb)

## Abbreviations

CBD: Cannabidiol
NASEM: The National Academies of Sciences, Engineering, and Medicine (NASEM)
QOL: Quality of life
THC: Tetrahydrocannabinol
