# Supplementary material for: A survey of the attitudes, beliefs and knowledge about medical cannabis among primary care providers
Source: BMC Fam Pract. 2019 Jan 22;20:17. doi: 10.1186/s12875-019-0906-y (PMC6341534; doi:10.1186/s12875-019-0906-y)
Supplement: Supplementary file 1 — Provider Cannabis Survey. Survey Tool Sent to Providers about Medical Cannabis. (DOCX 33 kb) [file 12875_2019_906_MOESM1_ESM.docx]

**Provider Cannabis Survey**

We appreciate your interest in completing the following survey. We are interested in the thoughts and beliefs that providers have about cannabis in medical practice.

Please review the following definitions which will help you in completing the survey.

**Cannabinoids** are substances coming from the plants *Cannabis sativa* or *Cannabis indica* (also know as “marijuana”) and are used for medical purposes or recreationally.

**Medical cannabis** refers to cannabinoids being made available through licensed medical centers only to individuals who have a doctor’s recommendation to possess and use cannabinoids to treat a medical condition.

1. Are you registered to certify patients for medical cannabis in the **Minnesota Cannabis Program?** Yes/no
2. Have you ever certified a patient for the **Minnesota Cannabis Program**? Yes/no
   1. If no, do you have any patients who have been certified?
3. If yes, approximately how many patients have you certified? (drop down)

Q1. Please indicate your agreement or disagreement with the following statements.

|  | Strongly agree (1) | Somewhat agree (2) | Neither agree nor disagree (3) | Somewhat disagree (4) | Strongly disagree (5) |
| --- | --- | --- | --- | --- | --- |
| Medical cannabis is a legitimate medical therapy. |  |  |  |  |  |
|  |  |  |  |  |  |
| Medical providers should be offering medical cannabis for managing medical conditions. |  |  |  |  |  |
|  |  |  |  |  |  |
| Medical cannabis has significant interactions with medical therapies. |  |  |  |  |  |
|  |  |  |  |  |  |
| Medical cannabis can effectively treat symptoms associated with medical conditions. |  |  |  |  |  |
|  |  |  |  |  |  |
| The process to certify patients in the medical cannabis program is difficult to navigate. |  |  |  |  |  |
|  |  |  |  |  |  |
| The process to certify patients in the medical cannabis program prevents me from enrolling patients. |  |  |  |  |  |
| I believe my patients not enrolled in the medical cannabis program are using cannabis illegally to treat their medical conditions. |  |  |  |  |  |

Q2 How helpful do you think cannabinoids are for the following symptoms:

|  | Very helpful (1) | Somewhat helpful (2) | Neither helpful nor not helpful (3) | Somewhat not helpful (4) | Not at all helpful (5) | Don’t know |
| --- | --- | --- | --- | --- | --- | --- |
|  |  |  |  |  |  |  |
| Pain |  |  |  |  |  |  |
| Seizures |  |  |  |  |  |  |
| Nausea and/or vomiting |  |  |  |  |  |  |
| Loss of appetite |  |  |  |  |  |  |
| Muscle spasms |  |  |  |  |  |  |
| Anxiety |  |  |  |  |  |  |
| Depression |  |  |  |  |  |  |
| Insomnia |  |  |  |  |  |  |
| Weight loss |  |  |  |  |  |  |
| Tics |  |  |  |  |  |  |

Q3. How helpful do you think that cannabinoids are for the treatment of the following conditions:

|  | Very helpful (1) | Somewhat helpful (2) | Neither helpful nor not helpful (3) | Somewhat not helpful (4) | Not at all helpful (5) | Don’t know |
| --- | --- | --- | --- | --- | --- | --- |
|  |  |  |  |  |  |  |
| - Cancer associated with severe/chronic pain, nausea or severe vomiting, or cachexia or severe wasting. |  |  |  |  |  |  |
| - Glaucoma. |  |  |  |  |  |  |
| - HIV/AIDS. |  |  |  |  |  |  |
| - Tourette Syndrome. |  |  |  |  |  |  |
| - Amyotrophic Lateral Sclerosis (ALS). |  |  |  |  |  |  |
| - Seizures, including those characteristic of Epilepsy. |  |  |  |  |  |  |
| - Severe and persistent muscle spasms, including those characteristic of Multiple Sclerosis. - Inflammatory bowel disease, including Crohn’s disease. - Terminal illness, with a probable life expectancy of less than one year* - [Intractable pain](http://www.health.state.mn.us/topics/cannabis/intractable/index.html) - Post-Traumatic Stress Disorder |  |  |  |  |  |  |
| - Obstructive Sleep apnea |  |  |  |  |  |  |
| - Autism |  |  |  |  |  |  |

Q3. To what extent do you think cannabis increases the risk for:

|  | A lot (1) | Somewhat (2) | A little (3) | Not at all | Don’t know |
| --- | --- | --- | --- | --- | --- |
|  |  |  |  |  |  |
| Psychotic symptoms |  |  |  |  |  |
| Depression |  |  |  |  |  |
| Memory problems |  |  |  |  |  |
| Respiratory symptoms |  |  |  |  |  |
| Accidents |  |  |  |  |  |
| Low birth weight |  |  |  |  |  |
| Drug overdose |  |  |  |  |  |
| Stroke |  |  |  |  |  |
| Diabetes |  |  |  |  |  |
| Heart attack |  |  |  |  |  |
| Cancer |  |  |  |  |  |

Q. To what extent **can medical cannabis** improve the following for patients:

|  | A great deal | Quite a bit | Somewhat | Very Little | Not at all | Don’t know |
| --- | --- | --- | --- | --- | --- | --- |
|  |  |  |  |  |  |  |
| Physical functioning |  |  |  |  |  |  |
| Energy level |  |  |  |  |  |  |
| Mood |  |  |  |  |  |  |
| Enjoyment of life |  |  |  |  |  |  |
| Social engagement (visiting with friends and family) |  |  |  |  |  |  |
| Ability to work |  |  |  |  |  |  |
| Sense of hope |  |  |  |  |  |  |

|  | A great deal | Quite a bit | Somewhat | Very Little | Not at all | I don’t’ want to answer questions about cannabis |
| --- | --- | --- | --- | --- | --- | --- |
|  |  |  |  |  |  |  |
| How prepared are you to answer patient questions about medical cannabis? |  |  |  |  |  |  |

1. Would you be interested in learning more about medical cannabis? Yes/no

| 1. Are you a: | |
| --- | --- |
| - 1. Physician (MD/DO/MBBS) (1) | |
| - 1. NP/PA (2) | |
| - 1. Other, please specify: (3) ____________________ |  |
|  |  |
| 1. What is your specialty:    1. Internal Medicine    2. Family Medicine 2. What is your age? |  |
|  |  |
| 1. How many years have you been in practice (since graduation for NP/PA, or since residency/fellowship completion for physicians)?  (drop down whole years only) |  |
|  |  |
| 1. What is your gender? |  |
| - 1. Male (1) |  |
| - 1. Female (2) |  |
|  |  |
